# Assessing cost-effectiveness of hepatitis C testing pathways in Georgia using the Hep C Testing Calculator

**DOI:** 10.1038/s41598-021-00362-y

**Published:** 2021-11-01

**Authors:** Madeline Adee, Yueran Zhuo, Huaiyang Zhong, Tiannan Zhan, Rakesh Aggarwal, Sonjelle Shilton, Jagpreet Chhatwal

**Affiliations:** 1 Massachusetts General Hospital, Boston, MA USA; 2 Harvard Medical School, Boston, MA USA; 3 College of Business, Mississippi State University, Mississippi State, MS USA; 4 Jawaharlal Institute of Postgraduate Medical Education and Research, Puducherry, India; 5 Foundation for Innovative New Diagnostics, Geneva, Switzerland

**Keywords:** Hepatitis C, Health care economics

## Abstract

The cost of testing can be a substantial contributor to hepatitis C virus (HCV) elimination program costs in many low- and middle-income countries such as Georgia, resulting in the need for innovative and cost-effective strategies for testing. Our objective was to investigate the most cost-effective testing pathways for scaling-up HCV testing in Georgia. We developed a Markov-based model with a lifetime horizon that simulates the natural history of HCV, and the cost of detection and treatment of HCV. We then created an interactive online tool that uses results from the Markov-based model to evaluate the cost-effectiveness of different HCV testing pathways. We compared the current standard-of-care (SoC) testing pathway and four innovative testing pathways for Georgia. The SoC testing was cost-saving compared to no testing, but all four new HCV testing pathways further increased QALYs and decreased costs. The pathway with the highest patient follow-up, due to on-site testing, resulted in the highest discounted QALYs (124 QALY more than the SoC) and lowest costs ($127,052 less than the SoC) per 10,000 persons screened. The current testing algorithm in Georgia can be replaced with a new pathway that is more effective while being cost-saving.

## Introduction

Chronic hepatitis C virus (HCV) infection is a global health problem that affects about 71 million people worldwide^1^. Of these, only 19% knew their infection status in 2017^1^. In many countries, HCV-related disease burden and deaths have been steadily increasing, despite recent advances in HCV treatment^1^. The highly effective direct-acting antivirals (DAAs) that became available from 2015 onwards can achieve high rates of sustained virologic response (SVR), a surrogate for cure^2^. However, a huge majority—more than 80%—of HCV patients remain undiagnosed and therefore are unable to avail the benefits of improved survival and quality of life provided by DAAs^1^.

The World Health Organization (WHO) recently launched a global strategy for elimination of HCV as a public health threat by the year 2030. This strategy aims to reduce HCV incidence by 80% and HCV-related mortality by 65%^3^. To reach this goal, the WHO estimates that by 2030 at least 90% of people with HCV need to be diagnosed, with a treatment rate of at least 80% among all treatment-eligible people with HCV^3^.

However, most countries do not have an HCV elimination strategy. In particular, for low- and middle-income countries (LMIC), which have limited resources but high HCV prevalence rates^4^, it is important to develop a cost-effective HCV elimination strategy. Given that the price of DAAs is low in most LMICs, the cost of testing can be a substantial contributor to the cost of HCV elimination^5^.

Georgia, a LMIC country, has a high HCV disease burden with prevalence of 5.4% in adults^6,7^, and has launched a national program to eliminate HCV. The Georgian health care system is largely private, but the national HCV elimination program formed a partnership between private and public institutions with a cost sharing model—with treatment provided for free through a donation from Gilead^8^. However, a recent study concluded that to achieve the goal of eliminating HCV as a public health threat in Georgia, innovative, simple, and cost-effective strategies are needed to scale-up HCV testing^9,10^. To help address this issue, the Foundation for Innovative Diagnostics (FIND) has proposed new testing pathways for HCV in Georgia.

The objective of this study was to evaluate the long-term cost-effectiveness of different HCV testing pathways in Georgia. We also developed an interactive online tool to assess and compare the health-related and economic outcomes of different pathways under different settings of HCV epidemic, patient flow and costs.

## Methods

### Overview

We utilized a state-transition model, MATCH (Markov-based Analyses of Treatments for Chronic Hepatitis C), which simulates HCV disease progression. Natural history outcomes from this model have been validated previously^11–13^. We adapted this model to simulate the epidemiology of HCV in Georgia (MATCH-Georgia), and extended the model to evaluate the cost-effectiveness of several innovative HCV testing pathways for Georgia. The model was developed following the principles on economic analyses with respect to viral hepatitis recommended by the WHO^14^. Using the results from this model, we also developed an interactive online tool, the Hep C Testing Calculator (www.hepccalculator.org), that allows users to compare the cost-effectiveness of different testing pathways for Georgia by entering key model inputs as applicable to the local situation.

### Baseline population characteristics

We ran the model for a general population cohort of 10,000 adults in Georgia, with an HCV antibody prevalence of 2% in the base case^6^, and the percentage of viremic infection among HCV antibody positive people of 75%. The baseline characteristics of HCV patients were determined by the different combinations of sex, HCV genotype, and METAVIR fibrosis stage observed in HCV patients in Georgia (Table 1). All HCV-infected patients were considered treatment-naïve because treatment coverage, until recently, had been very limited in Georgia. We assumed an average baseline age of 45 years. No human subjects were involved in this research.

**Table 1:** Baseline population characteristics among HCV-infected persons in Georgia.

Parameter	Value
**Age (median), years**^15^	39
**Fibrosis score distribution**^6^	
F0	38%
F1	32%
F2	13%
F3	10%
F4	7%
**Sex distribution**	
Male	50%
Female	50%
**Virus genotype distribution**^6,7,16^*	
G1	40%
G2	24%
G3	34%
G4	2%

*HCV* hepatitis C virus, *F* METAVIR fibrosis score, *G* genotype.

*HCV genotypes 5 and 6 were not considered because of their rarity in Georgia.

All the distributions in this table, including fibrosis score, sex and genotype were taken as independent of each other and assumed to have no dependencies.

### Testing pathways

**Figure 1:**
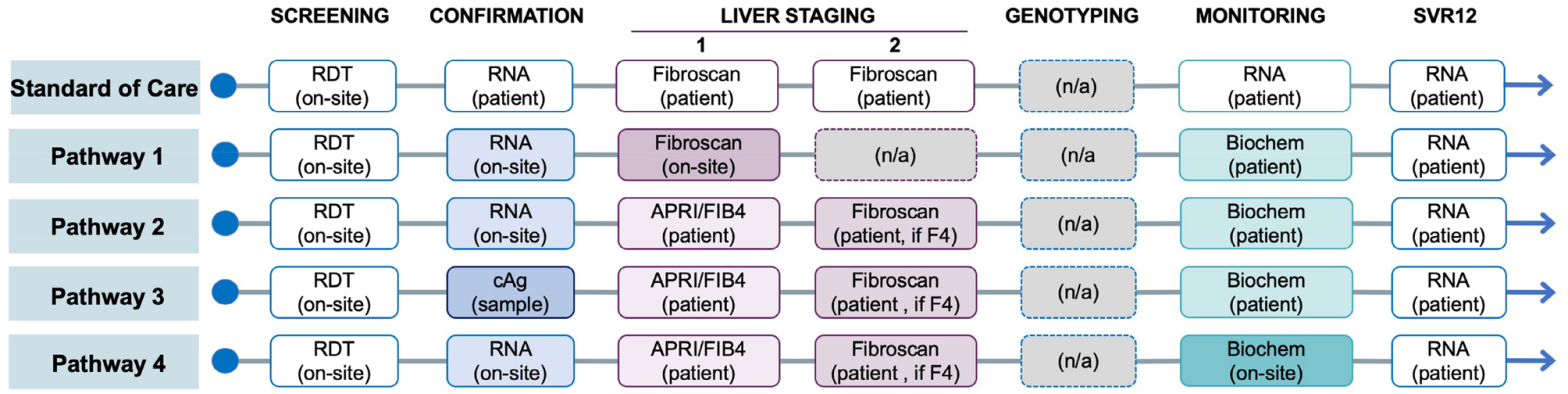
Patient flow under the current standard-of-care testing pathway and innovative hepatitis C testing pathways in Georgia. Abbreviations: RDT, rapid diagnostic test; RNA, ribonucleic acid test; cAg, core antigen test; APRI/FIB4, aspartate aminotransferase (AST)-to-platelet ratio index (APRI)/fibrosis-4 index; F4, METAVIR fibrosis score of 4.

We simulated five testing pathways for HCV diagnosis and monitoring. Among these pathways, one represents the standard of care (SoC) in Georgia, whereas the other four represent innovative testing pathways proposed by FIND and initiated under the HEAD-Start Harm Reduction study^17^. Each pathway consists of several sequential testing stages including initial screening, confirmation of presence of HCV RNA, liver staging, and treatment response (Fig. 1). Pathways differ in the testing technologies used (including sensitivity and specificity of each test) and in locations where each test is performed—on-site, specimen collected on-site and then sent to a laboratory, or at another location that the patient must travel to. All pathways use on-site HCV-antibody rapid diagnostic testing for screening. Confirmation is done using either HCV-RNA testing or HCV core-antigen testing. Liver staging consists of two phases of testing using either Fibroscan or APRI/FIB4, and for some pathways phase 2 is completed only for patients with METAVIR fibrosis score of 4. Treatment is monitored using RNA or biochemical testing, and all pathways use RNA testing for SVR evaluation.

### DAA treatment regimens and efficacy

Patients with viremic HCV infection who made it through the second liver-staging test in the pathway (or the first, if only one was done) were eligible to receive DAA-based treatment. The DAA regimens used in the model were determined by individual patient’s liver fibrosis stage. Data about the regimens, including their efficacy in different scenarios, were obtained from clinical trials^18–20^ (Supplementary Table S1).

### Disease progression

**Figure 2:**
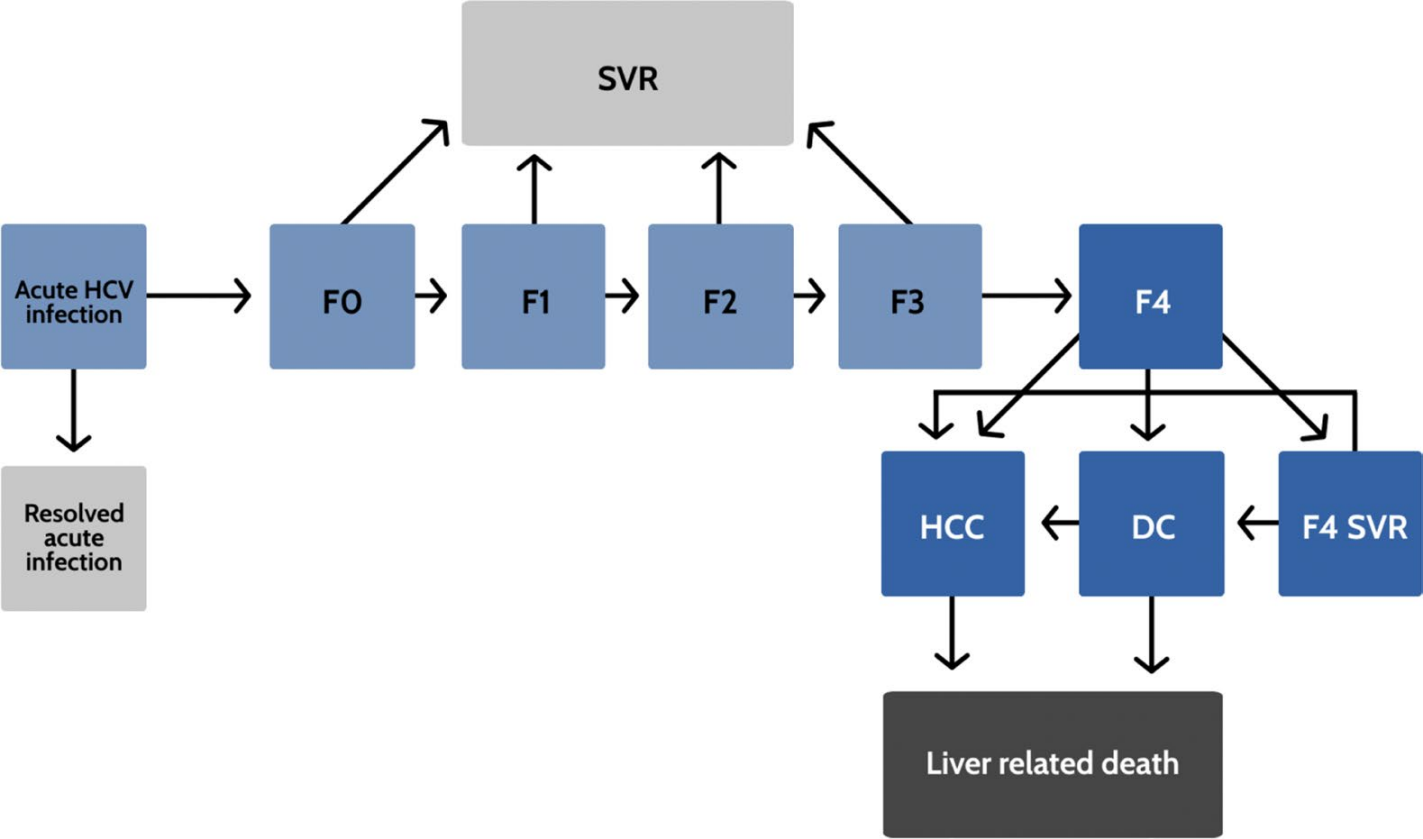
Model schematic of the natural history of hepatitis C virus in MATCH-Georgia model. Abbreviations: SVR, sustained virologic response; F0–F4, METAVIR fibrosis score; DC, decompensated cirrhosis; HCC, hepatocellular carcinoma; F4-SVR, sustained virologic response achieved at fibrosis stage 4.

Patients with HCV followed the natural history of HCV disease progression, defined as Markov health states in the MATCH-Georgia model (Fig. 2). Each patient started in a METAVIR liver fibrosis state of F0–F4. At the end of each simulation cycle (defined as one week), the patient could remain in the same state, progress into a more severe adjacent state of fibrosis, decompensated cirrhosis (DC), hepatocellular carcinoma (HCC), or liver-related death (LRD) or background mortality. Patients in the F0–F3 states who achieved SVR were considered cured and followed background mortality from that point on. However, patients who achieved SVR in the F4 state could still progress to DC, HCC and LRD states, though at a lower rate than F4 patients who had not achieved SVR^21^. Patients who fail treatment were assumed to continue to progress at the original rate. The fibrosis progression rates from F0 to F4 were based on a published meta-regression analysis^22^; progression rates from cirrhosis to DC and HCC are from published observational studies^23,24^. The liver-related mortality rates from DC and HCC were also derived from a published study^25^. The model did not include liver transplantation as a state due to the rarity of this procedure in Georgia.

### Quality of life weights

The model assigns quality-of-life (QoL) weights to each health state. All HCV-related QoL weights were derived from published studies^26–28^. People without HCV were assigned QoL weights according to their sex and age, and for patients who achieved SVR, the QoL weights of the health states were assumed to be equivalent to that of the non-HCV-infected general population^26^. However, if patients who achieved SVR progressed to DC or HCC, then the QoL weights of the corresponding state was applied. The adverse effect of anemia on quality of life during the treatment period was also considered, by applying an anemia multiplier. All HCV-related and normal QoL weight values are summarized in Table 2. The use of QoL weights allows us to present health-related outcomes as quality-adjusted life years (QALYs).

**Table 2:** Model parameters used in the MATCH-Georgia model.

Parameter Name	Base Case	Low	High	Distribution
**Testing pathway parameters (costs in USD)**				
Antibody RDT test unit cost^29^	1.00	0.50	1.50	Gamma (18, 0.0556)
HCV-RNA on-site test unit cost (Genexpert)^29^	15.00	8.00	23.00	Gamma (17, 0.8824)
APRI test unit cost^29^	5.00	3.00	8.00	Gamma (14, 0.3571)
Fibroscan test unit cost^29^	33.00	17.00	50.00	Gamma (17, 1.9412)
Biochemical test unit cost^29^	10.00	5.00	15.00	Gamma (19, 0.5263)
Genotyping test unit cost^29^	51.00	26.00	77.00	Gamma (18, 2.8333)
HCV-RNA test by referral unit cost^29^	40.00	20.00	60.00	Gamma (18, 2.22222)
Core antigen (cAg) test unit cost^29^	12.00	6.00	18.00	Gamma (19, 0.6316)
Sample transportation cost^29^	0.37	0.19	0.56	Gamma (18, 0.0206)
Cost of treatment^30^	100.00	50.00	150.00	Gamma (19, 5.2632)
HCV antibody prevalence^6^	2%	1%	3%	Beta (32, 1568)
Viremic rate in antibody-positive people	75%	50%	100%	Beta (19, 6.3333)
Target screening rate (assumption)	90%	75%	100%	Beta (38, 4.22222)
Confirmation test follow-up rate (Expert opinion^29^)	90%	75%	100%	Beta (38, 4.22222)
Liver staging-1 test follow-up rate (Expert opinion^29^)	90%	75%	100%	Beta (38, 4.22222)
Liver staging-2 test follow-up rate (Expert opinion^29^)	90%	75%	100%	Beta (38, 4.22222)
Monitoring test follow up-rate (Expert opinion^29^)	90%	75%	100%	Beta (38, 4.22222)
SVR12 RNA test follow-up rate (Expert opinion^29^)	90%	75%	100%	Beta (38, 4.22222)
**Transition probabilities (annual)**				
F0 to F1^22^	0.117	0.104	0.130	Beta (285.98,2158.26)
F1 to F2^22^	0.085	0.075	0.096	Beta (239.77, 2581)
F2 to F3^22^	0.120	0.109	0.133	Beta (351.88, 2580.45)
F3 to F4^22^	0.116	0.104	0.129	Beta (304.4, 2319.73)
F4 to DC^23^	0.039	0.010	0.079	Beta (4.87, 120.08)
F4 to HCC^23^	0.014	0.010	0.079	Beta (0.64, 44.75)
Post F4-SVR to DC^21^	0.008	0.002	0.036	Beta (0.87, 107.97)
Post F4-SVR to HCC^21^	0.005	0.002	0.013	Beta (3.28, 653.57)
DC to HCC^24^	0.068	0.030	0.083	Beta (24.48, 335.51)
DC (year 1) to death from liver disease^24^	0.182	0.065	0.190	Beta (27.56, 123.89)
DC (1 + years) to death from liver disease^24^	0.112	0.065	0.190	Beta (11.29, 89.55)
HCC to liver-related death^23^	0.427	0.330	0.860	Beta (5.52, 7.41)
Health state costs (annual in USD)*				
F0–F2	62	31	123	Gamma (6, 10.3333)
F3	126	63	253	Gamma (5, 25.2)
Compensated cirrhosis	144	72	289	Gamma (6, 21)
Decompensated cirrhosis	1496	748	2993	Gamma (17, 88)
Hepatocellular cancer	2625	1413	5652	Gamma (17.17, 154.4118)
F4 post-SVR	72	36	144	Gamma (5,14.4)
**Health state quality-of-life weights**				
Anemia multiplier^31^	0.83	0.75	0.97	Beta (80, 16.3855)
F0–F3^26^	0.93	0.84	1.00	Beta (40, 3.0108)
Compensated cirrhosis (F4)^26^	0.90	0.81	0.99	Beta (50, 5.5556)
DC^26^	0.80	0.57	0.99	Beta (12, 3)
HCC^26^	0.79	0.54	0.99	Beta (10, 2.6582)
Post-SVR***	1	0.92	1	Beta (3833.92, 3.84)
**Test sensitivity and specificity**				
Antibody RDT sensitivity^32^	98.0%	98.0%	100.0%	Uniform (0.98, 1)
Antibody RDT specificity^32^	100.0%	100%	100.0%	Uniform (1, 1)
HCV-RNA (lab) test sensitivity	99.8%	99.6%	100.0%	Uniform (0.996, 1)
HCV-RNA (lab) test specificity	99.7%	99.4%	100.0%	Uniform (0.994, 1)
cAg (lab) test sensitivity^33^	93.4%	90.10%	96.40%	Beta (150, 10.5996)
cAg (lab) test specificity^33^	98.8%	97.40%	99.50%	Beta (150, 1.8219)
**Sex and age-based normal health utility values**^26^				
Female, age < 29	0.913	–	–	–
Female, age 30–39	0.893	–	–	–
Female, age 40–49	0.863	–	–	–
Female, age 50–59	0.837	–	–	–
Female, age 60–69	0.811	–	–	–
Female, age 70–75	0.711	–	–	–
Male, age < 29	0.928	–	–	–
Male, age 30–39	0.918	–	–	–
Male, age 40–49	0.887	–	–	–
Male, age 50–59	0.861	–	–	–
Male, age 60–69	0.840	–	–	–
Male, age 70–75	0.802	–	–	–

*RDT* rapid diagnostic tests, *RNA* ribonucleic acid confirmation test, *APRI* aspartate aminotransferase to platelet ratio test, *FIB4* fibrosis-4 test, *cAg* core antigen test, *SVR* sustained virologic response, *F0–F4* METAVIR fibrosis score, *DC* decompensated cirrhosis, *HCC* hepatocellular carcinoma, *F4-SVR* sustained virologic response achieved at fibrosis stage 4.

*We estimated annual healthcare costs associated with HCV disease management using the World Health Organization’s CHOosing Interventions that are Cost Effective (WHO-CHOICE) tool.

** For patients experienced anemia during treatment, quality of life was multiplied by this factor.

***For patients who achieved SVR, the QoL weights of the health states are assumed to be equivalent to that of the non-HCV-infected general population^26^. For patients who achieve SVR at state F4 but further progressed to DC and HCC, their QoL weights were adjusted to those of DC and HCC, respectively.

### Costs

The MATCH-Georgia model considered HCV diagnosis costs, DAA-based treatment costs and HCV disease management costs. All costs were considered from a healthcare payer’s perspective.

The HCV testing costs consisted of the cost incurred at each step of the testing pathway, which included not only the costs for conducting laboratory tests, but also those for specimen shipment or of patient travel, as required, as these are integral parts of each testing pathway (Table 2). These costs were estimated from FIND HEAD-Start Georgia study^29^. Patients who failed to follow up in the next testing stage no longer incurred any further testing costs and did not receive DAA treatment. Free DAA medicines are now available for Georgia HCV patients through contracts with the pharmaceutical companies^30^, however we added $100 per person treated as an operational expense.

We estimated annual healthcare costs associated with HCV disease management using the World Health Organization’s CHOosing Interventions that are Cost Effective (WHO-CHOICE) tool^34^ (Table 2). For that, we first extracted inpatient and outpatient primary costs from WHO-CHOICE and took the weighted average of cost per inpatient visit and cost per outpatient visit for each HCV-associated states in the United States; inpatient visits accounted for 38% of healthcare encounters for F0–F4 patients, 43% for compensated cirrhosis patients, 66% for DC patients, and 55% for HCC patients^35^. We then estimated the ratio of the above costs in Georgia to United States and, finally, estimated Georgia-specific costs by multiplying this ratio with costs in the United States^35^. To account for differences in medical practices between Georgia and the United States, we considered a wide range in costs in the sensitivity analysis.

### Model outcomes

For each pathway, we projected average QALYs, total cost, and cumulative incidence of DC, HCC, and HCV-related deaths. We also estimated the testing costs per case treated and incremental cost-effectiveness ratio (ICER) of each pathway. A lifetime horizon was used, and all future costs and QALYs were discounted at 3% per year.

### Interactive tool

We also developed an interactive online tool using R Shiny that allows users to change certain inputs and evaluated the comparative effectiveness and cost-effectiveness of different diagnostic testing pathways. In this tool, users can change the population cohort size, screening rate, prevalence rates (of anti-HCV antibody in the population and of viremia among HCV-seropositive persons), and patient/client follow up rate for each step in a testing pathway—with the ability to add custom testing pathways. The users can also change costs for DAAs, each diagnostic test, patient/client travel, and specimen shipment.

Once parameters are changed, the tool shows updated results for the total expected QALYs, costs, and disease burden for each of the testing pathways. It also calculates the ICERs of the testing pathways by comparing their QALYs and costs, to assist users in identifying the most cost-effective testing pathways. A screenshot of the interactive tool is provided in Supplemental Figure S1. The tool is still being expanded and can be accessed at hepccalculator.org.

### Sensitivity analysis

We performed both deterministic and probabilistic sensitivity analyses to evaluate the effect of variations in model inputs on the cost-effectiveness of the testing pathways. These inputs included state transition probabilities, QoL weights, medical and disease management costs, diagnostic test costs, patient travel/sample shipping costs, and patient follow-up rates. Both the one-way and probabilistic sensitivity analyses also included HCV demographic parameters such as HCV prevalence and viremic rate in HCV antibody positive people. The ranges of all model inputs used for sensitivity analyses, and distribution used for the probabilistic sensitivity analysis, are defined in Table 2.

## Results

### Cost-effectiveness of HCV testing pathways

Compared with no screening, HCV screening under the SoC increased discounted QALYs by 332 per 10,000 people screened and decreased costs by US $290,942 (Table 3). All the four new HCV testing pathways (*Pathways 1–4*; Fig. 1) further increased QALYs and decreased costs. *Pathway 1*—on-site rapid diagnostic test for HCV antibody followed by on-site HCV-RNA confirmatory test, on-site Fibroscan for liver disease staging of chronic HCV patients, sample transportation for genotype testing, and on-site HCV-RNA test for assessment of treatment response—resulted in the highest discounted QALYs of 205,702 (124 QALY more than that under the SoC) and lowest costs of $142,939 ($127,052 less than that under SoC) per 10,000 persons screened. Compared with other pathways, *Pathway 1* was cost-saving. The testing-related cost per HCV case treated for the SoC was $289 and for *Pathway 1* was $139. Pathways 2, 3, and 4 all had higher total costs as well as higher testing costs per patient treated.

**Table 3:** Comparison of health-related outcomes and economic outcomes of the five screening pathways vs. no screening per 10,000 persons screened.

	No screening	Standard of care	Pathway 1	Pathway 2	Pathway 3	Pathway 4
**Total cost**	$560,933	$269,991	$142,939	$225,122	$251,769	$225,389
Disease management	$560,933	$233,067	$111,080	$196,638	$220,262	$196,638
Testing	_	$27,053	$18,315	$17,516	$21,250	$17,783
Treatment	_	$9,871	$13,544	$10,968	$10,257	$10,968
**QALYs (total cohort)**	205,246	205,578	205,702	205,615	205,591	205,615
**% viremic diagnosed**	0.0%	79.2%	88.0%	88.0%	82.4%	88.0%
**% viremic treated**	0.0%	64.2%	88.0%	71.3%	66.7%	71.3%
**Testing cost per treated pt**	_	$281	$139	$164	$213	$166
**No. needed to screen to diagnose one HCV case**		84	76	76	81	76
**No. needed to screen to prevent one LRD**		556	333	456	526	456
**Disease Burden**						
Decompensated cirrhosis	48	20	9	17	19	17
Hepatocellular carcinoma	30	13	6	11	12	11
Liver-related deaths (LRD)	41	23	11	19	22	19

*DC* decompensated cirrhosis, *HCC* hepatocellular carcinoma, *LRD* HCV-caused liver related death.

*The cost for no screening represents the cost of management of HCV sequelae.

### Clinical efficacy of testing pathways

The diagnosis rate—defined as the percentage of people with viremic HCV who were eventually diagnosed—of the SoC was 79.2%; by contrast, the diagnosis rate of *Pathway 1* was 88%. Patients lost before initiating treatment accounted for a bigger difference between the percent of viremic patients treated, with only 64.2% of viremic patients treated in the SoC scenario but 88% of viremic patients treated in *Pathway 1*. Under SoC, 84 people needed to get antibody screening on average to diagnose one additional HCV-viremic case, while under *Pathway 1* this number was 76. All new HCV testing pathways improved the HCV diagnosis rate.

The new pathways also improved clinical outcomes. Compared with the SoC, screening 10,000 people under *Pathway 1* would reduce the number of DC cases by 11, HCC by 7, and liver-related deaths by 12 in the lifetime horizon. The number of people needed to be screened (for antibody) to avoid one liver-related death for the SoC was 556, for *Pathway 1* was 333, for *Pathway 2* and *Pathway 4* was 456, and for *Pathway 3* was 526.

### Sensitivity analyses

**Figure 3:**
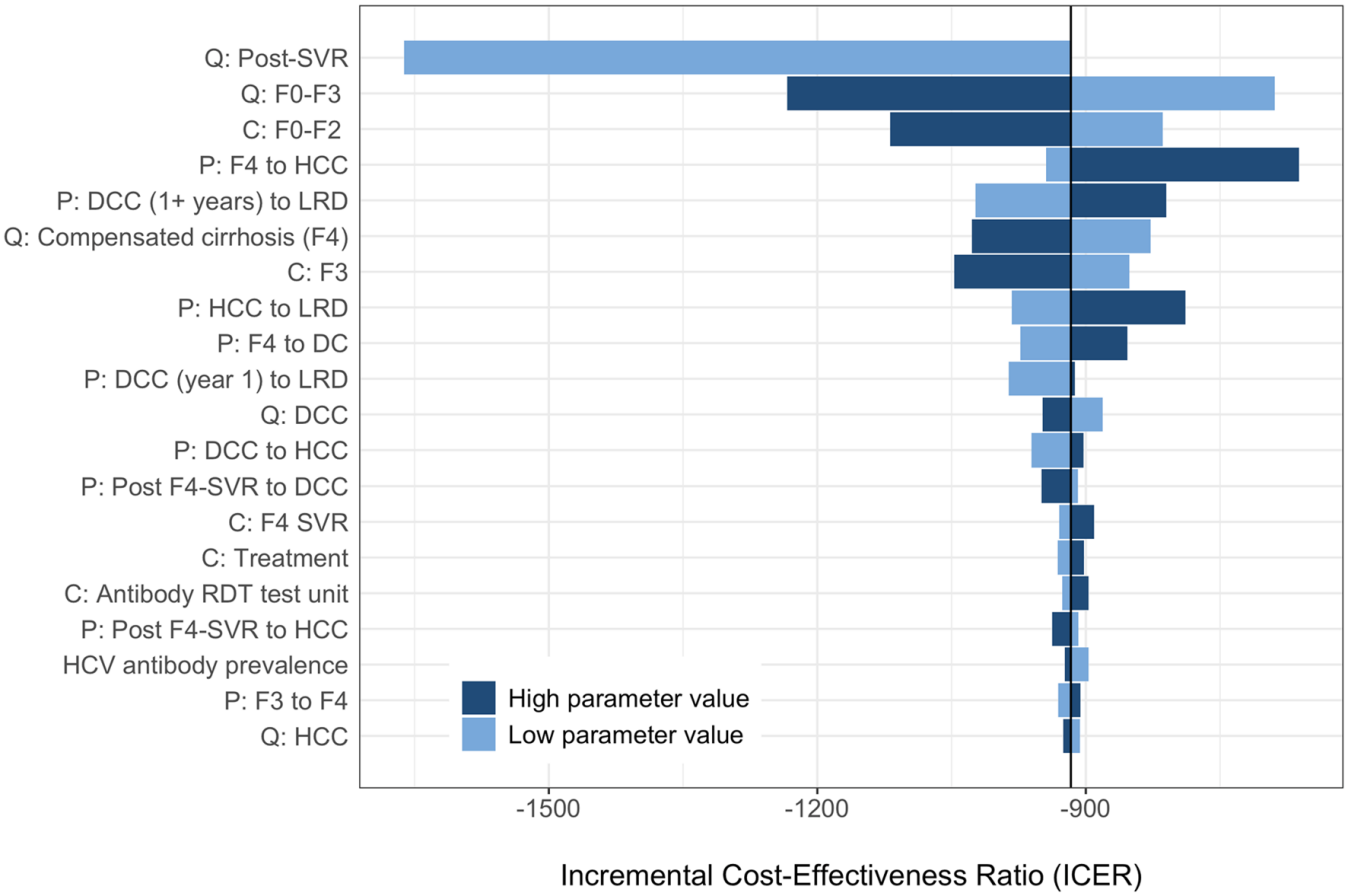
Tornado diagram for one-way sensitivity analysis of incremental cost-effectiveness ratio of *Pathway 1* versus *no screening* strategy. Horizontal bars show the variation in incremental cost-effectiveness ratio (ICER; in USD/QALY) with variation in the value of the parameter. In the parameter names, the prefix ‘C’ represents cost of a health-state, ‘Q’ the quality-of-life weight and ‘P’ the transition probability from one state to the other. Values of ICER below 0 indicate that the treatment is cost-saving. Abbreviations: SVR, sustained virologic response; F0–F4, METAVIR fibrosis score; DC, decompensated cirrhosis; HCC, hepatocellular carcinoma; F4-SVR, LRD, liver related death.

**Figure 4:**
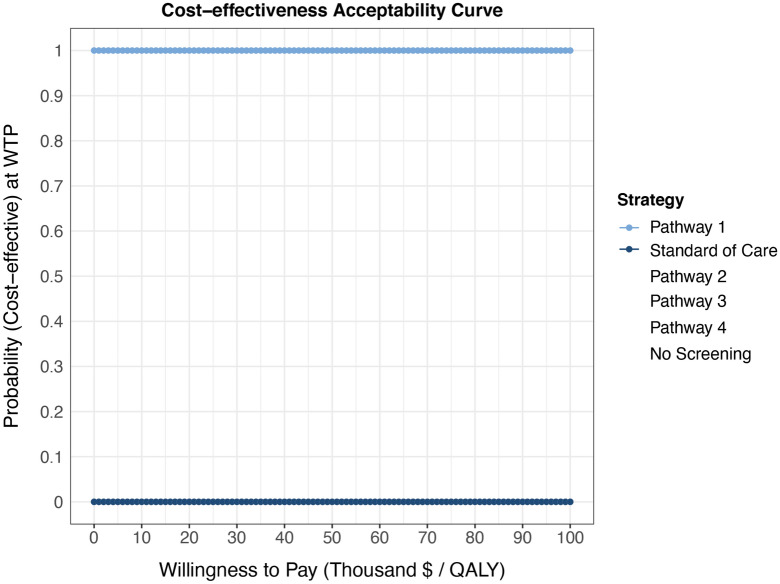
Cost-effectiveness Acceptability Curve of all pathways and *no screening* strategy.

*Pathway 1* remained cost-saving irrespective of the changes in model parameters. Figure 3 shows the 20 parameters that the model is most sensitive to, including QoL after achieving SVR, QoL of patients in F1-F4 states, probability of disease progression from F4 to DC, and costs of managing DC and HCC. One-way sensitivity analysis results for all parameters are shown in Supplement Table S2. Parameters related to the testing pathways, such as costs of different tests or of patient travel or sample shipping and patient/client follow-up rates had less marked influence on the cost-effectiveness of the testing pathways. For the probabilistic sensitivity analysis, Pathway 1 is the preferred cost-saving option in all scenarios, which is illustrated by the cost-effectiveness acceptability curve (Fig. 4).

## Discussion

The availability of highly effective yet low-priced HCV treatment in LMIC offers an unprecedented opportunity to eliminate HCV as a public health threat. However, the majority of HCV patients remain undiagnosed and hence are not in a position to avail the benefits of new treatments. In this study, we evaluated the cost-effectiveness of five different testing pathways to diagnose and monitor HCV during treatment in Georgia. We found that the pathway using on-site HCV-antibody rapid diagnostic test and HCV-RNA testing, followed by on-site Fibroscan was cost-saving—this pathway would save US $127,052 per 10,000 individuals tested (compared with the current standard), while increasing rates of diagnosis and linkage to successful treatment. This pathway would cost $139 per HCV case treated and could diagnose 88% of the viremic cases if scaled-up at the population level.

As pointed out by a recent study, substantial scaling-up of HCV testing and treatment are needed to eliminate HCV in Georgia^9,10^. However, that study did not evaluate what testing strategies would be cost-effective in Georgia. Therefore, our study fills an important evidence gap. We found that the preferred cost-effective strategies may depend on locally-determined factors, such as the HCV disease epidemiology, costs of different testing methodologies, patient follow-up rates following each visit or procedure, and the on-site availability of diagnostics such a Fibroscan and genotyping, leading to need for patient or specimen transport. We therefore also developed an interactive online-based tool that allows users to change several parameters in the model and identify the cost-effective testing pathway for their localized settings.

As the cost of DAAs has fallen below $100 per treatment in Georgia (and other LMICs), the diagnostic cost per HCV case constitutes a substantial portion of HCV care expenses. The cost of diagnosing one HCV case exceeds the cost of HCV treatment in Georgia, which could also be true for many LMICs where low-cost DAAs are available. All countries must domestically finance for these HCV testing and treatment efforts, as there is no global funding mechanism for HCV elimination. This contrasts with HIV, TB, and malaria, for which the Global Fund provides substantial budget annually^36^. Hence, it is very important for LMICs to identify HCV testing pathways that are cost-effective or cost-saving.

Interactive models such as this diagnosis pathway tool and our previously-developed *Hep C Treatment Calculator*^13^ are important tools to help aid countries, in particular LMICs, in understanding how to best use their existing domestic resources. Since the epidemiology of HCV varies geographically, having a tool that can be fed with location-specific epidemiology and cost inputs could provide countries with the context-specific cost-effectiveness estimates needed for their decision making. Our interactive tool takes this one step further. Even within a country, delivery of HCV services to different population groups may require different modes of service provision^37^. This tool can aid in tailoring testing pathway approaches that programs may seek to implement to reach various groups, such as PWID, MSM, age cohorts, regional groups, and others, with differing HCV prevalence and viremia rates, and cost of delivery of each test in specific settings. Hence, our tool could help the public health community to identify and implement the most effective and cost-effective strategy in different settings.

Lastly, it is important to note that the lost-to-follow-up rate remains an important consideration for country-level decision makers and program managers. Our analysis shows that the lost-to-follow-up rate has a limited impact on the incremental cost-effectiveness ratio when comparing several testing pathways—however this could be because the pathways have similar set-ups in terms of follow-up rate. An increase in lost-to-follow-up rates will have a similar negative impact on all pathways simultaneously. However, our analysis does not diminish the importance of the lost-to-follow-up rate in HCV testing practice, but rather shows that this issue needs addressing irrespective of the testing pathway chosen.

Our study has some limitations. First, our analysis did not account for continued HCV transmission. Therefore, the benefits of HCV testing, which serves to guide treatment and cure leading to reduced risk of transmission, could be even higher and the optimal pathway could result in even higher cost-savings. Second, since Georgia-specific QoL weights are not available, we used QoL weights from other countries. Our analysis also does not account for different QoL or mortality for specific populations within Georgia, such as people who inject drugs. However, sensitivity analysis suggests that the results remain robust to a wide range of input parameters and that QoL estimates did not change the conclusion of the study. Third, we note that the cost of DAAs in Georgia is negligible due to the contract made with major pharmaceutical companies, a situation that does not apply to most other countries.

In conclusion, our study identified a novel testing pathway to diagnose HCV and monitor its treatment in Georgia with greater effectiveness and found that such a testing pathway would result in cost-savings over the SoC pathway. Our online interactive tool can provide optimal HCV testing pathway under different settings of HCV epidemiology, costs of different tests, patient follow-up rates, and the on-site availability of diagnostics.

## Supplementary Information

Supplementary Information.
