# Author Correction: Assessing cost-effectiveness of hepatitis C testing pathways in Georgia using the Hep C Testing Calculator

**DOI:** 10.1038/s41598-022-07001-0

**Published:** 2022-02-17

**Authors:** Madeline Adee, Yueran Zhuo, Huaiyang Zhong, Tiannan Zhan, Rakesh Aggarwal, Sonjelle Shilton, Jagpreet Chhatwal

**Affiliations:** 1 Massachusetts General Hospital, Boston, MA USA; 2 Harvard Medical School, Boston, MA USA; 3 College of Business, Mississippi State University, Mississippi State, MS USA; 4 Jawaharlal Institute of Postgraduate Medical Education and Research, Puducherry, India; 5 Foundation for Innovative New Diagnostics, Geneva, Switzerland

Correction to: *Scientific Reports* 10.1038/s41598-021-00362-y, published online 01 November 2021

The original version of this Article contained errors in the Abstract and Results section.

In the Abstract,

“The pathway with the highest patient follow-up, due to on-site testing, resulted in the highest discounted QALYs (123 QALY more than the SoC) and lowest costs ($127,052 less than the SoC) per 10,000 persons screened.”

now reads:

“The pathway with the highest patient follow-up, due to on-site testing, resulted in the highest discounted QALYs (124 QALY more than the SoC) and lowest costs ($127,052 less than the SoC) per 10,000 persons screened.”

In the Results section, under the subheading ‘Cost-effectiveness of HCV testing pathways’,

“Compared with no screening, HCV screening under the SoC increased discounted QALYs by 333 per 10,000 people screened and decreased costs by US $290,942 (Table 3). All the four new HCV testing pathways (*Pathways 1–4*; Fig. 1) further increased QALYs and decreased costs. *Pathway 1*—on-site rapid diagnostic test for HCV antibody followed by on-site HCV-RNA confirmatory test, on-site Fibroscan for liver disease staging of chronic HCV patients, sample transportation for genotype testing, and on-site HCV-RNA test for assessment of treatment response—resulted in the highest discounted QALYs of 169,753 (123 QALY more than that under the SoC) and lowest costs of $142,939 ($127,052 less than that under SoC) per 10,000 persons screened.”

now reads:

“Compared with no screening, HCV screening under the SoC increased discounted QALYs by 332 per 10,000 people screened and decreased costs by US $290,942 (Table 3). All the four new HCV testing pathways (*Pathways 1–4*; Fig. 1) further increased QALYs and decreased costs. *Pathway 1*—on-site rapid diagnostic test for HCV antibody followed by on-site HCV-RNA confirmatory test, on-site Fibroscan for liver disease staging of chronic HCV patients, sample transportation for genotype testing, and on-site HCV-RNA test for assessment of treatment response—resulted in the highest discounted QALYs of 205,702 (124 QALY more than that under the SoC) and lowest costs of $142,939 ($127,052 less than that under SoC) per 10,000 persons screened.”

In addition, the Article contained errors in Table 3, where the QALYs (total cohort) values were incorrect for “No screening”, “Standard of care”, “Pathway 1”, “Pathway 2”, “Pathway 3” and “Pathway 4”. The incorrect and correct values appear below.

Incorrect:

**Table:** 

	No screening	Standard of care	Pathway 1	Pathway 2	Pathway 3	Pathway 4
QALYs (total cohort)	169,297	169,630	169,753	169,666	169,643	169,666

Correct:

**Table:** 

	No screening	Standard of care	Pathway 1	Pathway 2	Pathway 3	Pathway 4
QALYs (total cohort)	205,246	205,578	205,702	205,615	205,591	205,615

The original Article has been corrected.

