# Supplementary material for: Assessing cost-effectiveness of hepatitis C testing pathways in Georgia using the Hep C Testing Calculator
Source: Sci Rep. 2021 Nov 1;11:21382. doi: 10.1038/s41598-021-00362-y (PMC8560949; doi:10.1038/s41598-021-00362-y)

**SUPPLEMENT**

**Assessing cost-effectiveness of hepatitis C testing pathways in Georgia using the Hep C Testing Calculator**

Madeline Adee^1¶^, Yueran Zhuo^1,2,3¶^, Huaiyang Zhong^1,2^, Tiannan Zhan^1^, Rakesh Aggarwal^4^, Sonjelle Shilton^5&^, Jagpreet Chhatwal^1,2&^ *

^1^Massachusetts General Hospital, Boston, Massachusetts, USA

^2^Harvard Medical School, Boston, Massachusetts, USA

^3^Mississippi State University College of Business, Mississippi State, Mississippi, USA

^4^Jawaharlal Institute of Postgraduate Medical Education and Research, Puducherry, India

^5^Foundation for Innovative New Diagnostics, Geneva, Switzerland

* Corresponding author

Email: [jagchhatwal@mgh.harvard.edu](mailto:jagchhatwal@mgh.harvard.edu)

^¶^ co-first authors, these authors contributed equally to this work

^&^ co-senior authors, these authors contributed equally to this work

**Table S1.** Treatment regimens used based on HCV genotype and patients’ liver fibrosis stage, and the corresponding expected rates of sustained virological response (SVR), treatment discontinuation and adverse events (AEs)

| **HCV genotype** | **METAVIR fibrosis stage** | **Treatment drugs** | **Treatment duration (weeks)** | **SVR (%)** | **Treatment discontinuation rate (%)** | **AE**  **(Anemia)**  **( %)** | **Duration of AEs (weeks)** |
| --- | --- | --- | --- | --- | --- | --- | --- |
| **G1** (15) | F0-F3 | SOF + LDV | 12 | 98.1 | 1 | 1 | 2 |
|  | F4 | SOF + LDV | 12 | 93.2 | 1 | 1 | 2 |
| **G2** (33, 34) | F0-F3 | SOF + RBV | 12 | 94.0 | 1 | 8 | 4 |
|  | F4 | SOF + RBV | 16 | 94.0 | 1 | 8 | 4 |
| **G3** (16, 35) | F0-F3 | SOF + DCV | 12 | 97.0 | 0 | 1 | 4 |
|  | F4 | SOF + DCV | 24 | 86.0 | 2 | 1 | 4 |
| **G4** (17) | F0-F4 | SOF + LDV | 12 | 95.0 | 1 | 2 | 2 |

Abbreviations: AE = adverse event, SOF = sofosbuvir, DCV = daclatasvir, LDV = ledipasvir

**Table S2.** One-way sensitivity analysis results. Results are shown only for Pathway 1 and No Screening, because in all scenarios tested in the sensitivity analysis Pathway 1 remains the lowest cost and highest QALY testing pathway option.

|  | **No Screening** | | | | **Pathway 1** | | | |
| --- | --- | --- | --- | --- | --- | --- | --- | --- |
|  | **Costs (USD)** | | **QALYS** | | **Costs (USD)** | | **QALYS** | |
| **Parameter** | **Low** | **High** | **Low** | **High** | **Low** | **High** | **Low** | **High** |
| C: Antibody RDT test unit | $ 560,933 | $ 560,933 | 169,297 | 169,297 | $ 138,439 | $ 151,939 | 169,753 | 169,753 |
| C: HCV-RNA on-site test unit | $ 560,933 | $ 560,933 | 169,297 | 169,297 | $ 141,182 | $ 144,947 | 169,753 | 169,753 |
| C: APRI test unit | $ 560,933 | $ 560,933 | 169,297 | 169,297 | $ 142,939 | $ 142,939 | 169,753 | 169,753 |
| C: Fibroscan test unit | $ 560,933 | $ 560,933 | 169,297 | 169,297 | $ 140,824 | $ 145,186 | 169,753 | 169,753 |
| C: Biochemical test unit | $ 560,933 | $ 560,933 | 169,297 | 169,297 | $ 142,345 | $ 143,533 | 169,753 | 169,753 |
| C: Genotyping test unit | $ 560,933 | $ 560,933 | 169,297 | 169,297 | $ 142,939 | $ 142,939 | 169,753 | 169,753 |
| C: HCV-RNA by referral test unit | $ 560,933 | $ 560,933 | 169,297 | 169,297 | $ 142,939 | $ 142,939 | 169,753 | 169,753 |
| C: Core antigen (cAg) test unit | $ 560,933 | $ 560,933 | 169,297 | 169,297 | $ 142,939 | $ 142,939 | 169,753 | 169,753 |
| C: Sample transportation | $ 560,933 | $ 560,933 | 169,297 | 169,297 | $ 142,939 | $ 142,939 | 169,753 | 169,753 |
| C: Treatment | $ 560,933 | $ 560,933 | 169,297 | 169,297 | $ 136,182 | $ 149,696 | 169,753 | 169,753 |
| HCV antibody prevalence | $ 280,466 | $ 841,399 | 169,299 | 169,296 | $ 75,970 | $ 209,909 | 169,527 | 169,980 |
| Viremic rate in Ab+ people | $ 373,955 | $ 747,910 | 169,298 | 169,296 | $ 98,301 | $ 187,577 | 169,602 | 169,904 |
| Target screening rate | $ 560,933 | $ 560,933 | 169,297 | 169,297 | $ 212,605 | $ 96,495 | 169,677 | 169,804 |
| Confirmation test follow-up rate | $ 560,933 | $ 560,933 | 169,297 | 169,297 | $ 142,939 | $ 142,939 | 169,753 | 169,753 |
| Liver staging-1 test follow-up rate | $ 560,933 | $ 560,933 | 169,297 | 169,297 | $ 142,939 | $ 142,939 | 169,753 | 169,753 |
| Liver staging-2 test follow-up rate | $ 560,933 | $ 560,933 | 169,297 | 169,297 | $ 142,939 | $ 142,939 | 169,753 | 169,753 |
| Monitoring test follow up-rate | $ 560,933 | $ 560,933 | 169,297 | 169,297 | $ 142,444 | $ 143,269 | 169,753 | 169,753 |
| SVR12 RNA test follow-up rate | $ 560,933 | $ 560,933 | 169,297 | 169,297 | $ 142,939 | $ 142,939 | 169,753 | 169,753 |
| Antibody RDT sensitivity | $ 560,933 | $ 560,933 | 169,297 | 169,297 | $ 142,939 | $ 134,225 | 169,753 | 169,763 |
| Antibody RDT specificity | $ 560,933 | $ 560,933 | 169,297 | 169,297 | $ 142,939 | $ 142,939 | 169,753 | 169,753 |
| HCV-RNA (lab) test sensitivity | $ 560,933 | $ 560,933 | 169,297 | 169,297 | $ 142,939 | $ 142,939 | 169,753 | 169,753 |
| HCV-RNA (lab) test specificity | $ 560,933 | $ 560,933 | 169,297 | 169,297 | $ 142,939 | $ 142,939 | 169,753 | 169,753 |
| cAg (lab) test sensitivity | $ 560,933 | $ 560,933 | 169,297 | 169,297 | $ 142,939 | $ 142,939 | 169,753 | 169,753 |
| cAg (lab) test specificity | $ 560,933 | $ 560,933 | 169,297 | 169,297 | $ 142,939 | $ 142,939 | 169,753 | 169,753 |
| P: F0 to F1 | $ 557,345 | $ 563,132 | 169,301 | 169,294 | $ 142,373 | $ 143,252 | 169,754 | 169,753 |
| P: F1 to F2 | $ 551,778 | $ 568,913 | 169,307 | 169,285 | $ 141,699 | $ 144,212 | 169,755 | 169,752 |
| P: F2 to F3 | $ 554,006 | $ 570,810 | 169,308 | 169,291 | $ 141,848 | $ 144,369 | 169,755 | 169,752 |
| P: F3 to F4 | $ 554,427 | $ 567,512 | 169,312 | 169,283 | $ 141,915 | $ 143,679 | 169,755 | 169,751 |
| P: F4 to DC | $ 465,263 | $ 625,728 | 169,427 | 169,181 | $ 128,016 | $ 152,812 | 169,773 | 169,735 |
| P: F4 to HCC | $ 557,634 | $ 569,586 | 169,317 | 169,076 | $ 142,564 | $ 143,915 | 169,756 | 169,719 |
| P: DCC to HCC | $ 576,105 | $ 555,813 | 169,308 | 169,294 | $ 145,965 | $ 141,799 | 169,755 | 169,752 |
| P: Post F4-SVR to DCC | $ 560,933 | $ 560,933 | 169,297 | 169,297 | $ 137,574 | $ 160,008 | 169,763 | 169,720 |
| P: Post F4-SVR to HCC | $ 560,933 | $ 560,933 | 169,297 | 169,297 | $ 141,604 | $ 145,997 | 169,759 | 169,740 |
| P: DCC (year 1) to LRD | $ 589,106 | $ 558,995 | 169,309 | 169,296 | $ 148,517 | $ 142,552 | 169,755 | 169,753 |
| P: DCC (1+ years) to LRD | $ 604,343 | $ 514,917 | 169,315 | 169,279 | $ 151,748 | $ 133,633 | 169,757 | 169,749 |
| P: HCC to LRD | $ 592,479 | $ 498,053 | 169,305 | 169,283 | $ 149,981 | $ 129,346 | 169,755 | 169,750 |
| C: F0-F2 | $ 506,064 | $ 668,901 | 169,297 | 169,297 | $ 134,830 | $ 158,897 | 169,753 | 169,753 |
| C: F3 | $ 525,851 | $ 630,528 | 169,297 | 169,297 | $ 137,757 | $ 153,218 | 169,753 | 169,753 |
| C: CC | $ 560,933 | $ 560,933 | 169,297 | 169,297 | $ 142,939 | $ 142,939 | 169,753 | 169,753 |
| C: DCC | $ 560,933 | $ 560,933 | 169,297 | 169,297 | $ 142,939 | $ 142,939 | 169,753 | 169,753 |
| C: HCC | $ 560,933 | $ 560,933 | 169,297 | 169,297 | $ 142,939 | $ 142,939 | 169,753 | 169,753 |
| C: F4 SVR | $ 560,933 | $ 560,933 | 169,297 | 169,297 | $ 137,042 | $ 154,734 | 169,753 | 169,753 |
| Q: Anemia multiplier | $ 560,933 | $ 560,933 | 169,297 | 169,297 | $ 142,939 | $ 142,939 | 169,753 | 169,753 |
| Q: F0-F3 | $ 560,933 | $ 560,933 | 169,118 | 169,437 | $ 142,939 | $ 142,939 | 169,725 | 169,776 |
| Q: Compensated cirrhosis (F4) | $ 560,933 | $ 560,933 | 169,239 | 169,355 | $ 142,939 | $ 142,939 | 169,744 | 169,762 |
| Q: DCC | $ 560,933 | $ 560,933 | 169,274 | 169,316 | $ 142,939 | $ 142,939 | 169,749 | 169,757 |
| Q: HCC | $ 560,933 | $ 560,933 | 169,291 | 169,302 | $ 142,939 | $ 142,939 | 169,752 | 169,754 |
| Q: Post-SVR | $ 560,933 | $ 560,933 | 169,297 | 169,297 | $ 142,939 | $ 142,939 | 169,549 | 169,753 |

**Figure S1.** Screenshot of the interactive tool.


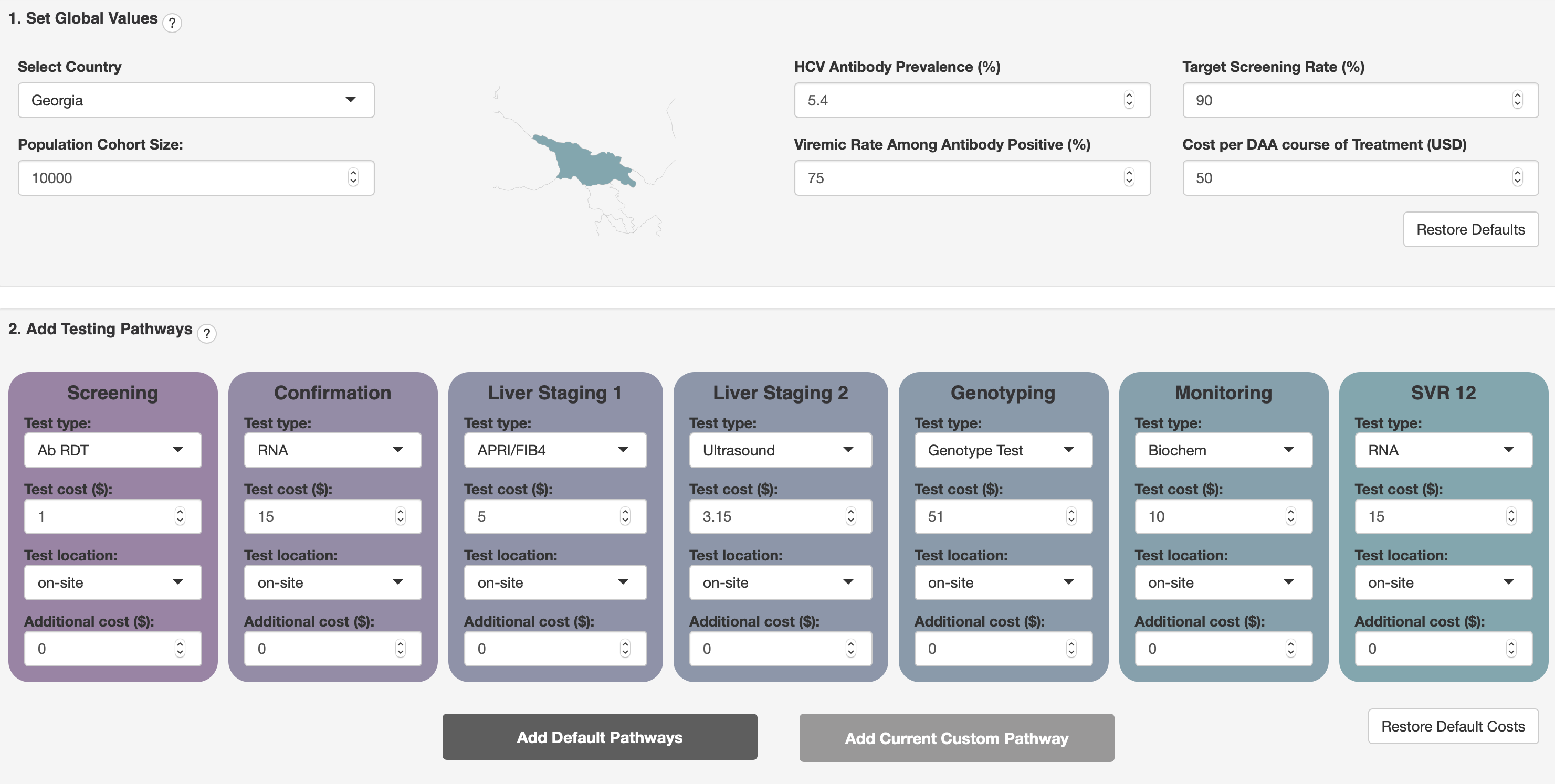

Supplement: Supplementary file 1 — Supplementary Information. [file 41598_2021_362_MOESM1_ESM.docx]
